# Molecular Characterisation of Small Molecule Agonists Effect on the Human Glucagon Like Peptide-1 Receptor Internalisation

**DOI:** 10.1371/journal.pone.0154229

**Published:** 2016-04-21

**Authors:** Aiysha Thompson, Jeffrey W. Stephens, Stephen C. Bain, Venkateswarlu Kanamarlapudi

**Affiliations:** Institute of Life Science 1, Medical School, Swansea University, Singleton Park, Swansea, United Kingdom; Dasman Diabetes Institute, KUWAIT

## Abstract

The glucagon-like peptide receptor (GLP-1R), which is a G-protein coupled receptor (GPCR), signals through both Gαs and Gαq coupled pathways and ERK phosphorylation to stimulate insulin secretion. The aim of this study was to determine molecular details of the effect of small molecule agonists, compounds 2 and B, on GLP-1R mediated cAMP production, intracellular Ca^2+^ accumulation, ERK phosphorylation and its internalisation. In human GLP-1R (hGLP-1R) expressing cells, compounds 2 and B induced cAMP production but caused no intracellular Ca^2+^ accumulation, ERK phosphorylation or hGLP-1R internalisation. GLP-1 antagonists Ex(9–39) and JANT-4 and the orthosteric binding site mutation (V36A) in hGLP-1R failed to inhibit compounds 2 and B induced cAMP production, confirming that their binding site distinct from the GLP-1 binding site on GLP-1R. However, K334A mutation of hGLP-1R, which affects Gα_s_ coupling, inhibited GLP-1 as well as compounds 2 and B induced cAMP production, indicating that GLP-1, compounds 2 and B binding induce similar conformational changes in the GLP-1R for Gα_s_ coupling. Additionally, compound 2 or B binding to the hGLP-1R had significantly reduced GLP-1 induced intracellular Ca^2+^ accumulation, ERK phosphorylation and hGLP-1R internalisation. This study illustrates pharmacology of differential activation of GLP-1R by GLP-1 and compounds 2 and B.

## Introduction

The glucagon like peptide-1 (GLP-1) hormone, which produced within the intestinal L-cells in response to food intake, is very effective in lowering blood glucose levels by increasing insulin secretion in type 2 diabetic patients [1–3]. GLP-1 exerts its actions through the GLP-1 receptor (GLP-1R), which is a member of the class B G-protein coupled receptor (GPCR) family [3–6]. GLP-1 is cleaved in secretory vesicles to form the bioactive peptides, GLP-1 (7–36)-NH_2_ and GLP-1 (7–37), bind to the GLP-1R with similar affinity and show similar potency [7,8]. *In vivo*, both the bioactive peptides of GLP-1 have a very short half-life (~1.5min) due to their rapid proteolytic degradation in plasma to GLP-1(9–36)-NH_2_ and GLP-1(9–37), respectively, by the dipeptidyl peptidase-IV (DPP-IV) [3].

Exendin-4, which is found in the saliva of the Gila monster lizard, also acts as an agonist to the GLP-1R [9, 10]. In contrast to the active forms of GLP-1, exendin-4 is resistant to proteolytic degradation by DPP-IV [11]. Truncated version of GLP-1 (GLP-1 [9–36]-NH_2_/[9–37]) and exendin-4 (exendin-3, Ex[9–39]) also bind to the GLP-1R but function as antagonists [9, 10, 12, 13]. Both GLP-1R agonists, liraglutide (a DPP-IV resistant GLP-1) and exenatide (a synthetic version of exendin-4), are currently in use as drugs for the treatment of patients with type 2 diabetes [14–16].

Small molecule agonists of the GLP-1R, compound 2 (6,7-dichloro-2-methylsulfonyl-3-*N*-*tert*-butylaminoquinoxaline) and compound B (4-(3-(benzyloxy)phenyl)-2-(ethylsulfinyl)-6-(trifluoromethyl)-pyramidine [BETP]), have also been developed [17, 18]. These compounds binding site(s) on GLP-1R is spatially and functionally distinct from the primary agonist GLP-1 (orthosteric) binding site [4, 19]. However, they act as ago-allosteric modulators of GLP-1R by enhancing GLP-1 binding to the GLP-1R [17, 18]. Consistent with this, compound 2 has been shown to potentiate significantly glucose induced insulin secretion in wild-type mouse islets but not in islets from the GLP-1R knockout mice [17]. Compound B has also been shown to induce near-normal insulin secretion in human islets isolated from a donor with type 2 diabetes [18]. Furthermore, compounds 2 and B act in an additive manner to increase GLP-1 induced insulin secretion [17, 18].

The agonist occupied GLP-1R signals through both the Gα_s_ and Gα_q_ coupled pathways [3, 5, 6]. The coupling of GLP-1R to the Gα_s_ pathway results in cyclic adenosine monophosphate (cAMP) production whereas the receptor coupling to the Gα_q_ pathway leads to intracellular calcium (Ca^2+^) accumulation and thereby the phosphorylation of extracellular signal-regulated kinase (ERK) [20]. Upon agonist binding, GLP-1R has been shown to rapidly internalise in a model cell line and mouse pancreatic islets to dampen the signal and recycle to resensitise the desensitised receptor [21]. We have recently shown that agonist-induced GLP-1R internalisation is mediated by the Gα_q_ pathway [20]. In addition, the C-terminus of GLP-1R plays an important role in agonist-induced internalisation of the receptor [22, 23].

The small molecule agonists, compounds 2 and B, have been shown to modulate differently the GLP-1R activation [24, 25]. However, the molecular details of the effect of compounds 2 and B on GLP-1R internalisation are not well characterised. In this study, the small molecule agonists, compounds 2 and B, on GLP-1R were pharmacologically assessed for their effects on human GLP-1R (hGLP-1R) mediated cAMP production, intracellular Ca^2+^ accumulation, ERK phosphorylation and internalisation of the receptor. We have also analysed pharmacologically whether compounds 2 and B bind to the GLP-1 binding site on hGLP-1R or not by using the GLP-1 antagonists Ex(9–39) [9, 10] and JANT-4 [26] and the hGLP-1R mutant V36A (defective in the orthosteric agonist binding). Furthermore, we assessed here the effect of compounds 2 and B on GLP-1 mediated GLP-1R activation and internalisation. We show that compounds 2 and B caused cAMP production, similar to that of GLP-1, in cells expressing hGLP-1R but induced neither intracellular Ca^2+^ accumulation nor ERK phosphorylation nor hGLP-1R internalisation. The antagonists Ex(9–39) and JANT-4 and the hGLP-1R V36A mutant abolished GLP-1 induced cAMP production but had no effect on cAMP production stimulated by compound 2 or compound B, confirming that they act as ago-allosteric modulators of GLP-1R. Further, we showed that the small molecule agonists inhibit GLP-1 induced hGLP-1R internalisation, intracellular Ca^2+^ accumulation and ERK phosphorylation. Taken together, these results suggest that ago-allosteric agonists such as compounds 2 and B binding to GLP-1R activate specific signalling pathways (biased agonism) that are less favourable to internalisation of the receptor.

## Materials and Methods

### Materials

The primary antibodies used were rabbit anti-vesicular stomatitis virus glycoprotein (VSVG) (Immunoblotting, ab34774, Abcam Biochemicals, Cambridge, UK), mouse anti-VSVG (ELISA, V5507, Sigma-Aldrich, Dorset, UK), mouse anti-green fluorescent protein (GFP) (11814460001, Roche, West Sussex, UK), mouse anti-GLP-1R (MAB2814, R&D Systems, Abington, UK), rabbit anti-phospho ERK1/2 (9102, pERK1/2) and rabbit ERK1/2 (9102, New England Biolabs, Hertfordshire, UK). The Cy3-conjugated anti-mouse immunoglobulin G (IgG) secondary antibody (715-165-150, Jackson Laboratories, Suffolk, UK) was used for immunofluorescence. Horseradish peroxidase (HRP)-conjugated anti-mouse (NA933) and anti-rabbit (NA934) IgG (GE Healthcare, Hertfordshire, UK) secondary antibodies were used for immunoblotting. Enhanced chemiluminescence (ECL) select reagent was obtained from GE Healthcare (Hertfordshire, UK). GLP-1 7–37 (liraglutide) was from Novo Nordisk (Sussex, UK). Compound 2, compound B and Ex(9–39) were purchased from Calbiochem (Nottingham, UK). JANT-4 was from Prof. Richard DiMarchi, Indiana University (IN, USA) [26]. All other chemicals were from Sigma-Aldrich (Dorset, UK) unless otherwise stated.

### Plasmids

The cDNA of SP-VSVG-hGLP-1RΔN23, containing the signal peptide (SP, 1–23 amino acids) coding sequence followed by VSVG coding sequence, was cloned into pEGFP-N1 vector (Clontech, Takara Bio Europe SAS, Saint-Germain-en-Laye, France), as described previously, for expression as the N-terminus VSVG-tagged and the C-terminus GFP-tagged fusion protein in mammalian cells (SP-VSVG-hGLP-1RΔN23-GFP). The V36A and K334A mutations within the hGLP-1R were generated using Quickchange II XL site-directed mutagenesis kit (Agilent Technologies, Leicestershire, UK) and SP-VSVG-hGLP-1RΔN23-GFP plasmid as the template [27]. Luciferase reporter plasmids pGL4.29-Luc-CRE, pGL4.30-Luc-NFAT and pGL4.33-Luc-SRE were from Promega (Southampton, UK).

### Cell culture and transfection

Human embryonic kidney (HEK)293 cells were maintained at 37°C in a 5% CO_2_ humidified environment in Dulbecco’s modified Eagle medium (DMEM; serum free medium [SFM]) supplemented with 10% foetal calf serum, 2mM glutamine, 100U/ml penicillin and 0.1mg/ml streptomycin (full serum medium [FSM]). Cells were transiently transfected for 48h using JetPrime transfection reagent (Polyplus; 2μl/μg DNA) according to the manufacturer’s instructions.

### Enzyme linked immunosorbent assay (ELISA)

This assay was carried out as described previously with unpermeabilised cells to quantify cell surface expression of the hGLP-1R [20, 28]. Briefly, HEK293 cells expressing the hGLP-1R were serum starved for 1h and then stimulated without or with agonist at 37°C/5% CO_2_. Where indicated, cells were incubated with antagonist for 30min prior and during stimulation with agonist at 37°C/5% CO_2_. Cells were then fixed with 4% paraformaldehyde (PFA,) for 10min, blocked with 1% BSA made in TBS (1% BSA/TBS) for 45min and probed with the anti-GLP-1R mouse antibody (diluted 1:15000) in 1% BSA/TBS for 1h. Cells were washed with TBS and then incubated with the HRP-conjugated anti-mouse IgG (diluted 1:5000) in 1% BSA/TBS for 1h. Cells were washed and developed using 1-step Ultra TMB-ELISA substrate (Bio-Rad, Herts, UK) for 15min and the reaction stopped by adding an equal volume of 2M sulphuric acid. The optical density was read at 450nm using a plate reader.

### Immunofluorescence

Intracellular localisation of hGLP-1R expression was assessed by immunofluorescence as described previously [20, 28]. Briefly, cells were serum starved for 1h and incubated without or with antagonist at the indicated concentration for 30min at 37°C/5% CO_2_ and then with the anti-GLP-1R mouse antibody (diluted 1:5000) in 1% BSA/DMEM for 1h at 4°C. Cells were stimulated without or with agonist in the absence or presence of antagonist at 37°C/5% CO_2_, fixed with 4% PFA for 30min, permeabilised with 0.2% Triton X-100 made in PBS for 10min, blocked in blocking buffer (1% BSA made in wash buffer [0.1% Triton X-100 in PBS]) for 30min and then incubated with the Cy3-conjugated anti-mouse antibody (diluted 1:200 in blocking buffer) for 1h. Cells were then washed 3 times with wash buffer and incubated with DAPI (4′,6-diamidino-2-phenylindole dihydrochloride, 1mg/ml) diluted 1:2000 in PBS to stain nucleus. Coverslips were mounted on glass microscopic slides using mounting solution (0.1M Tris-HCl, pH 8.5, 10% Mowiol 50% glycerol) containing 2.5% DABCO (1,4 diazabicyclo [2.2.2] octane). Immunofluorescence staining was visualised using Zeiss LSM710 confocal microscope fitted with a 63x oil immersion lens [27].

### Live cell fluorescence imaging

For live cell fluorescence imaging, HEK293 cells transiently transfected with SP-VSVG-hGLP-1RΔN23-GFP plasmid for 24h were plated into an 8 chamber glass bottom slide (Thermo Scientific) pre-coated with 0.1mg/ml poly-L-lysine and incubated at 37°C/5% CO_2_ in FSM. After 24h, cells were washed 3 times with and incubated in 250μl per well of SFM for 1h at 37°C/5% CO_2_ for serum starvation. Live cells were then imaged at 37°C by using Zeiss LSM710 confocal microscope fitted with a 63x oil immersion lens. Cells were imaged twice (0 and 3 min) before adding agonist and for every 3min after stimulating with agonist for 60min, as described previously [21, 29].

### cAMP assay

Cells were serum starved for 1h and then stimulated, in the presence of 0.25mM phosphodiesterase inhibitor Ro201724, without or with 100nM GLP-1 for 1h at 37°C/5% CO_2_. Cells were then lysed and cAMP levels in the cell lysates were estimated using the cAMP direct immunoassay kit (Abcam, Cambridge, UK), as described previously [20, 28].

### cAMP, Ca^2+^ and ERK luciferase assay

This assay was carried out as described [20]. HEK293 cells cotransfected with the plasmids of the hGLP-1R and luciferase reporter for cAMP (pGL4.29-Luc-CRE) or intracellular Ca^2+^ (pGL4.30-Luc-NFAT) or ERK phosphorylation (pGL4.33-Luc-SRE) were treated with increasing concentrations of agonist for 4h (cAMP and ERK) or 8h (Ca^2+^) at 37°C/5% CO_2_. After the stimulation, an equal volume of ONE-Glo^™^ lysis buffer containing luciferase substrate (Promega, Southampton, UK) was added to each well and luminescence measured using a plate reader in accordance with the manufacturer’s instructions.

### Cell lysates and immunoblotting

The cell lysates were prepared and used in immunoblotting as described previously [20, 28]. HEK293 cells expressing hGLP-1R were lysed in ice-cold modified RIPA lysis buffer (10mM Tris-HCl, pH 7.5, containing 10mM EDTA, 1% NP40, 0.1% SDS, 0.5% sodium deoxycholate and 150mM NaCl) with 1% complete mammalian protease inhibitor mixture. The cell lysates mixed with ½ volume of 3x SDS-PAGE sample loading buffer (75mM Tris HCl, pH 6.8, containing 3% SDS, 30% glycerol, 0.003% bromophenol blue and 0.3M dithiothreitol [DTT]) was incubated at room temperature for 1h and then used to detect hGLP-1R expression by immunoblotting using the anti-GFP and anti-VSVG antibodies [30, 31].

### Data analysis

Data were analysed using the GraphPad Prism programme. The data presented as mean ± SEM of three independent experiments. Statistical comparisons between a control and test value was made by a one-tailed paired student t-test. Statistical analysis between multiple groups was determined by the Bonferroni’s post test after one-way or two-way analysis of variance (ANOVA), where p>0.05 was considered as statistically not significant (n.s.), and p<0.05, p<0.01, and p<0.001 shown as *, ** and *** respectively. Concentration response curves were also fitted using Prism, according to a standard logistic equation. Confocal images of fixed cells shown in the figures are representative of 190–200 transfected cells from three different experiments. The confocal images of live cells shown in figures are representative of 3 independent cell preparations. Similarly, immunoblotting data shown in the figures are representative of three independent experiments.

## Results

### Pharmacological analysis of two small molecule agonists of the hGLP-1R

**Fig 1:**
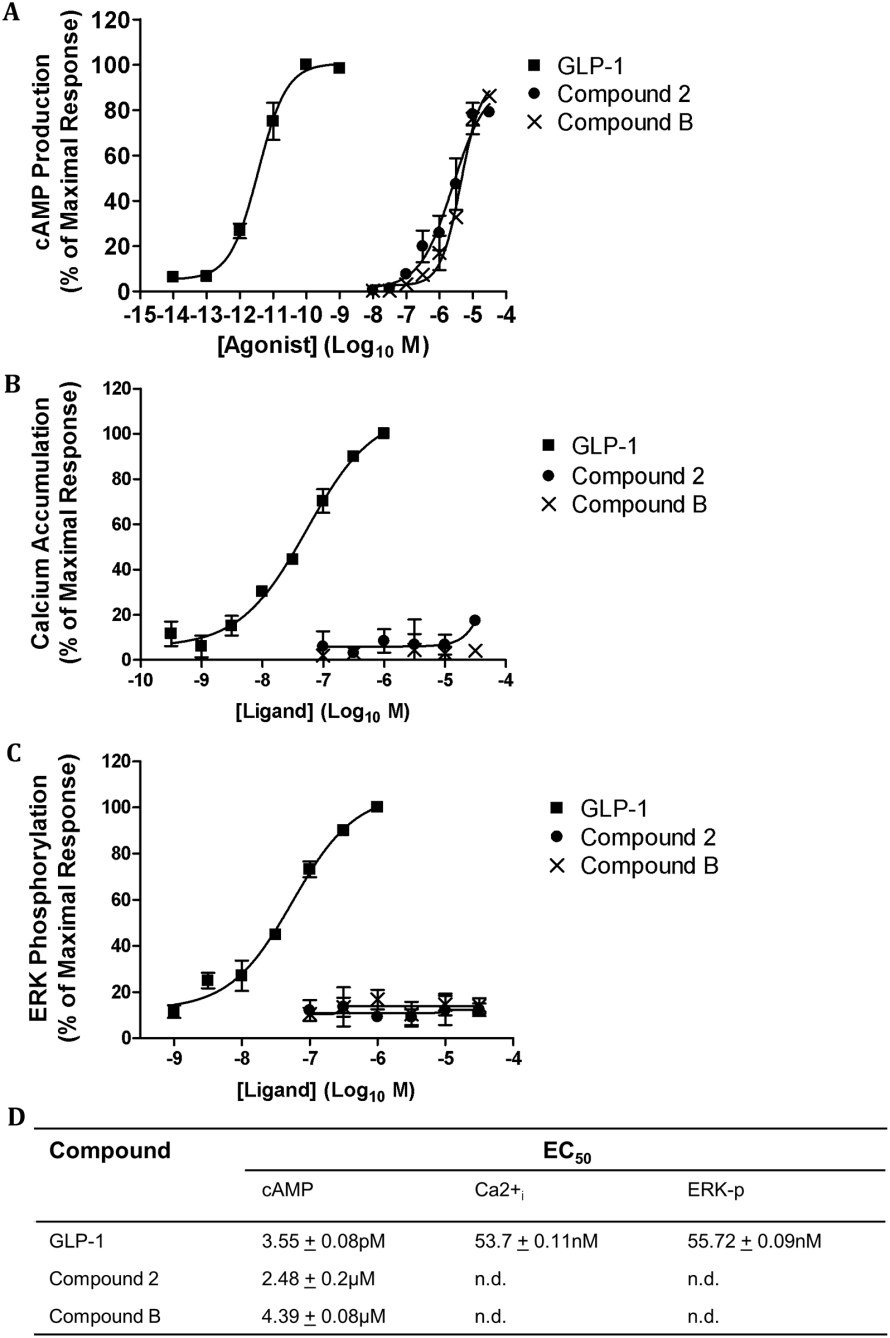
Small molecule agonists induce cAMP production but not intracellular Ca^2+^ accumulation or ERK phosphorylation in hGLP-1R expressing cells. HEK293 cells cotransfected with the hGLP-1R plasmid and the luciferase reporter plasmid for cAMP (pGL4.29-Luc-CRE), intracellular Ca^2+^ (pGL4.30-Luc-NFAT) or ERK phosphorylation (pGL4.33-Luc-SRE) were stimulated with GLP-1, compound 2 or compound B as indicated to assess cAMP production (A), intracellular Ca^2+^ accumulation (Ca^2+^_i_) (B) and ERK phosphorylation (ERK-p) (C). (D) The EC_50_ of GLP-1, compounds 2 and B for cAMP production, (Ca^2+^_i_) and ERK-p (n.d., not determined). Data normalised to percentage stimulation of GLP-1 and are shown as mean ± SEM, n = 3.

We have recently shown that two small molecule agonists (compounds 2 and B) of GLP-1R stimulate cAMP production but have no effect on GLP-1R internalisation, intracellular Ca^2+^ accumulation or ERK phosphorylation [20]. Here, compounds 2 and B agonistic effect on the hGLP-1R was pharmacologically assessed (using the cAMP production, intracellular Ca^2+^ accumulation, ERK phosphorylation and receptor internalisation as readouts (Fig 1)) and compared to that of GLP-1. GLP-1 stimulated a concentration dependent increase in cAMP production in HEK293 cells expressing the hGLP-1R (Fig 1A). Compound 2 and compound B also induced the same levels of cAMP production in hGLP-1R expressing HEK293 cells, demonstrating both compounds 2 and B stimulate cAMP production with E_max_ values similar to that of GLP-1 [25]. However, the EC_50_ of GLP-1 for inducing cAMP production is 3 orders of magnitude lower than that of compounds 2 and B (Fig 1D) [25]. GLP-1 also potentiated intracellular Ca^2+^ accumulation (Fig 1B) and ERK phosphorylation (Fig 1C) in a concentration dependent manner in hGLP-1R expressing cells. However, compounds 2 and B had no effect on intracellular Ca^2+^ accumulation and ERK phosphorylation. Taken together, these results demonstrate compounds 2 and B induce cAMP production with similar E_max_ to GLP-1 but do not potentiate intracellular Ca^2+^ accumulation or ERK phosphorylation in hGLP-1R expressing cells.

**Fig 2:**
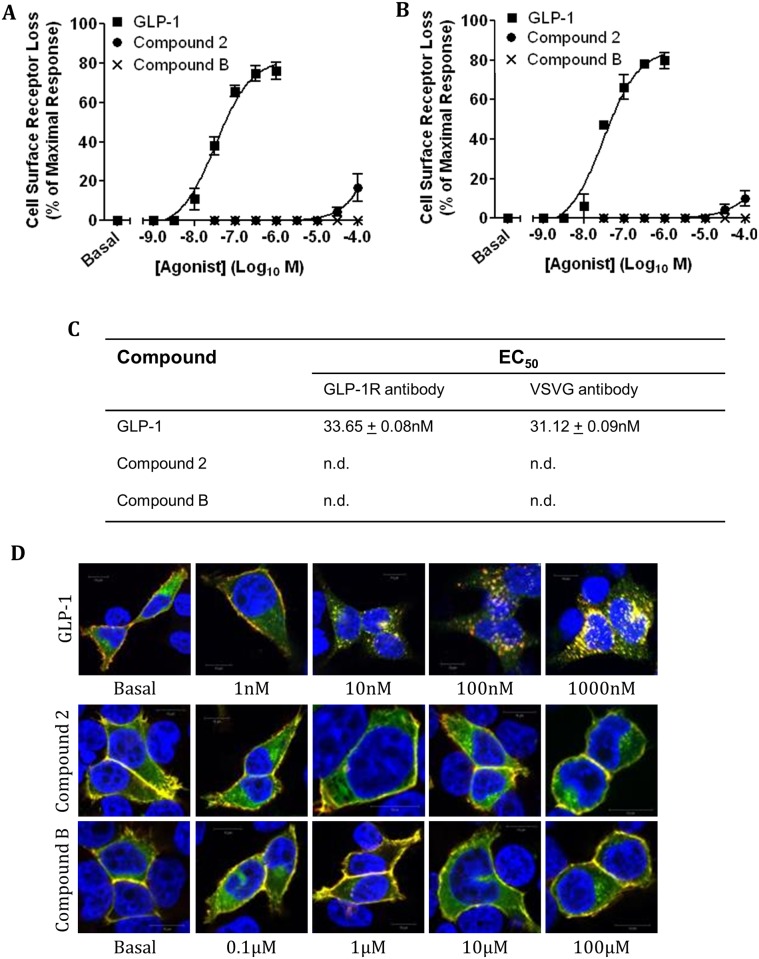
Concentration dependent stimulation of hGLP-1R internalisation by GLP-1, compound 2 and compound B. HEK293 cells expressing the hGLP-1R were stimulated with GLP-1, compound 2 or compound B at the indicated concentration for 60min and hGLP-1R internalisation was assessed by ELISA using the anti-GLP-1R antibody (A) and the VSVG-antibody (B) and by immunofluorescence (D). (C) The EC_50_ of GLP-1, compounds 2 and B for hGLP-1R internalisation (n.d., not determined). In immunofluorescence, GFP-tagged hGLP-1R (green) and the anti-GLP-1R antibody staining (red) overlay is shown in yellow and nuclear staining with DAPI in blue. Data are mean ± SEM, n = 3.

Since intracellular Ca^2+^ accumulation and ERK phosphorylation are required for GLP-1 stimulated hGLP-1R internalisation [20], the effect of compounds 2 and B on hGLP-1R internalisation was pharmacologically assessed next. HEK293 cells expressing the hGLP-1R were challenged with increasing concentrations of GLP-1, compound 2 or compound B for 60min and then the cell surface expression of the receptor was analysed by ELISA using the anti-GLP-1R antibody (Fig 2A) and the anti-VSVG antibody (Fig 2B). The orthosteric agonist GLP-1 induced a dose dependent increase in hGLP-1R internalisation and had a maximal effect of 76.03 ± 4.4% at 100nM. The EC_50_ of GLP-1 for hGLP-1R internalisation is shown in Fig 2C. Interestingly, compound 2 showed no induction of hGLP-1R internalisation up to 3.3μM and at its highest concentration (100μM) only 16.63 ± 6.96% of cell surface receptors were internalised. Additionally, compound B showed no effect on internalisation of the receptor up to a concentration of 100μM. We were unable to use higher than 0.1mM of compounds 2 and B since they found to be toxic to HEK293 cells above that concentration (data not shown) [4]. When hGLP-1R internalisation was assessed by ELISA using the anti-VSVG antibody, the results obtained were similar to that obtained with the anti-GLP-1R antibody (Fig 2B and 2C). This indicated the anti-GLP-1R antibody does not interfere with compound 2 and compound B binding to the receptor and therefore only the anti-GLP-1R antibody was used in further experiments. These results were confirmed by immunofluorescence analysis (Fig 2D) where intracellular punctate structures, indicative of hGLP-1R internalisation, were observed in cells treated with GLP-1, but were absent in cells treated with compound 2 or B.

**Fig 3:**
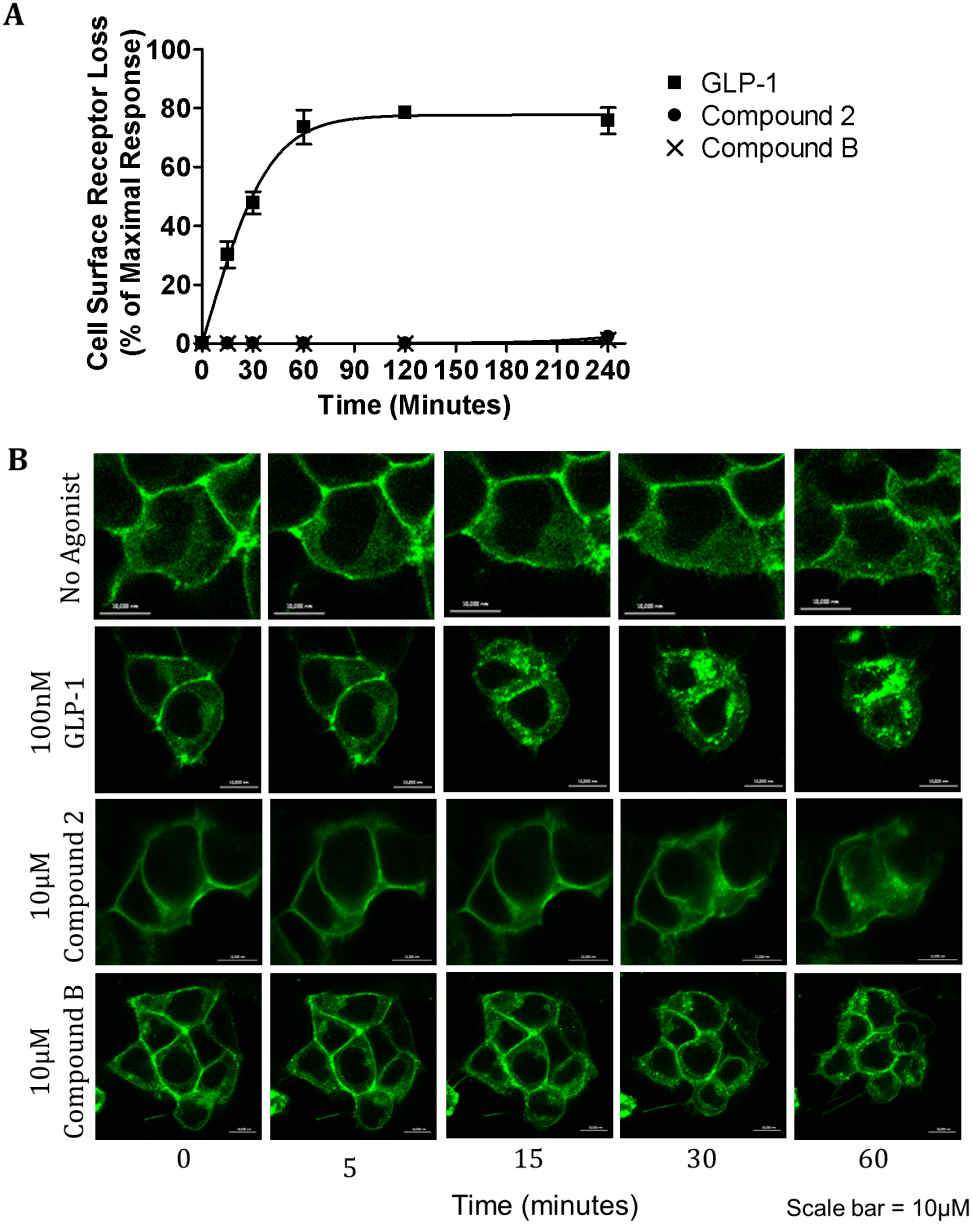
Time dependent stimulation of hGLP-1R internalisation by GLP-1, compound 2 and compound B. HEK293 cells expressing the hGLP-1R were stimulated with 100nM GLP-1, 10μM compound 2 or 10μM compound B for the indicated time and assessed hGLP-1R internalisation by ELISA using the anti-GLP-1R antibody (A) and live cell fluorescence imaging (B). Data are mean + SEM, n = 3 (A) or representative of three independent experiments (B).

Additionally, the time dependent effect of GLP-1, compound 2 and compound B on hGLP-1R internalisation was determined by using ELISA (Fig 3A) and live cell imaging (Fig 3B). GLP-1 induced hGLP-1R internalisation in a time dependent manner, reaching maximum internalisation of the receptor at approximately 60min of stimulation (73.57 ± 5.81%). In contrast, no internalisation of the receptor was observed for compounds 2 and B. Live cell fluorescence imaging showed the appearance of internalised cell surface GFP-tagged receptor as intracellular punctate structures when challenged with GLP-1 but not with compound 2 or compound B, supporting the ELISA results. Together, these results demonstrate that, unlike GLP-1, the small molecule agonists do not internalise the hGLP-1R most likely because they are unable to induce intracellular Ca^2+^ accumulation or ERK phosphorylation.

### Ex(9–39) and JANT-4 act as antagonists for GLP-1 but not compounds 2 and B

**Fig 4:**
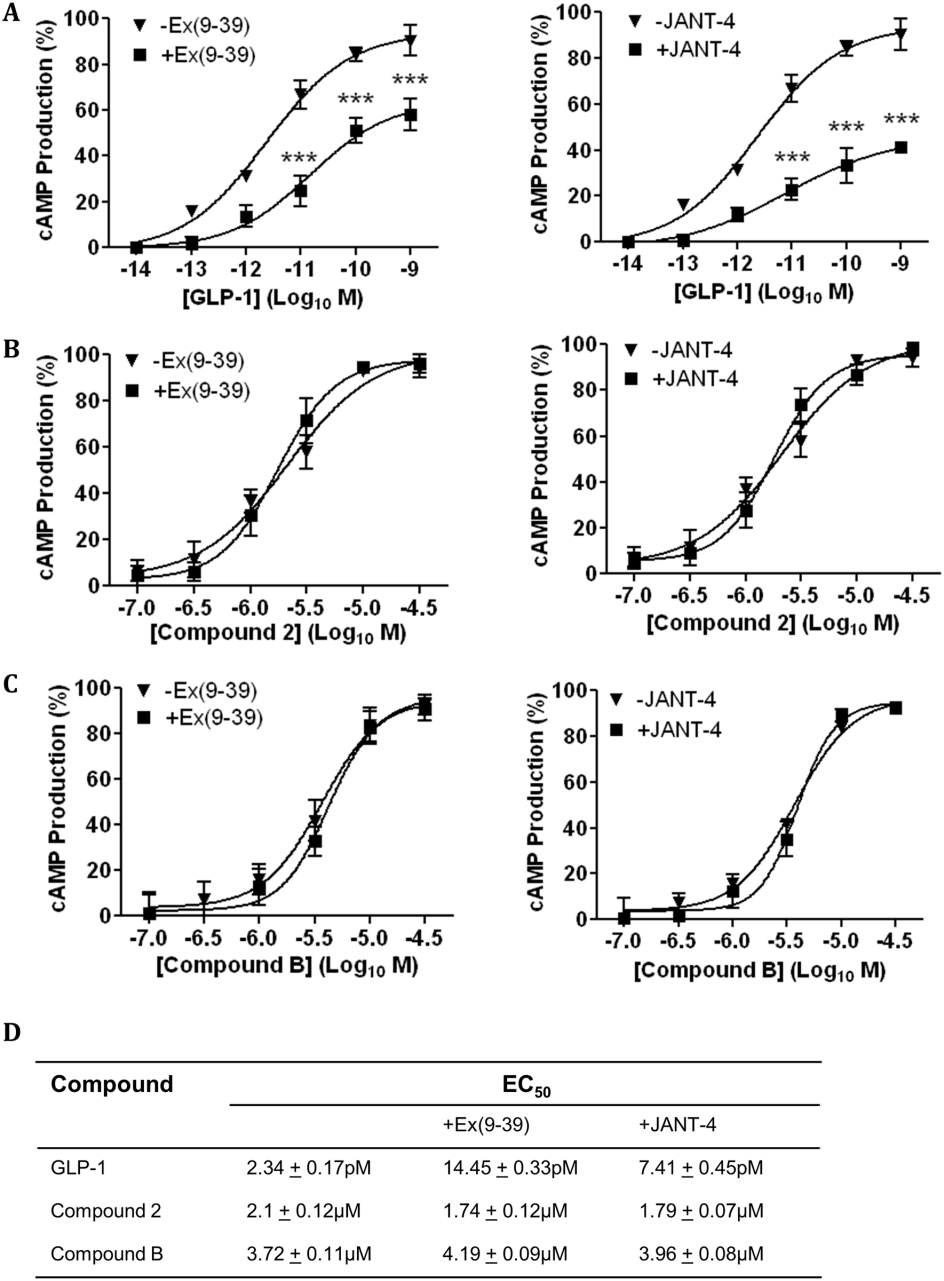
Antagonists Ex(9–39) and JANT-4 inhibit cAMP production induced by GLP-1 but not compound 2 or compound B. HEK293 cells cotransfected with the hGLP-1R and the luciferase reporter plasmid for cAMP (pGL4.29-Luc-CRE) were stimulated with GLP-1 (A), compound 2 (B) or compound B (C), as indicated, in the presence of 100nM Ex(9–39) (left panel) or JANT-4 (right panel) to assess cAMP production. (D) The EC_50_ of GLP-1, compounds 2 and B in the presence or absence of Ex(9–39) or JANT-4 for cAMP production. Data are mean ± SEM, n = 3, *** p <0.001.

Ex(9–39) and JANT-4 are known antagonist of the GLP-1R that work by binding to the orthosteric binding site [9, 10, 26, 32], which therefore competitively inhibit GLP-1 binding to the receptor. Compounds 2 and B have been described as ago-allosteric agonists [4, 17, 18, 33]. To confirm this, the effect of antagonists Ex(9–39) and JANT-4 on these small molecule agonists was determined. For this purpose, hGLP-1R expressing cells pre-incubated with Ex(9–39) or JANT-4 were stimulated with GLP-1 (Fig 4A), compound 2 (Fig 4B) or compound B (Fig 4C) and then determined cAMP production. The EC_50_ of GLP-1, compound 2 and compound B in the presence and absence of Ex(9–39) or JANT-4 for cAMP production is shown in Fig 4D. GLP-1 stimulated a dose dependent increase in cAMP production. In the presence of Ex(9–39) or JANT-4, GLP-1 induced cAMP production was reduced. In contrast, Ex(9–39) and JANT-4 had no effect on compound 2 stimulated cAMP production. Similarly, antagonists Ex(9–39) and JANT-4 had no effect on the cAMP production stimulated by compound B. These results confirmed compound 2 and compound B do not bind to the orthosteric agonist binding site.

**Fig 5:**
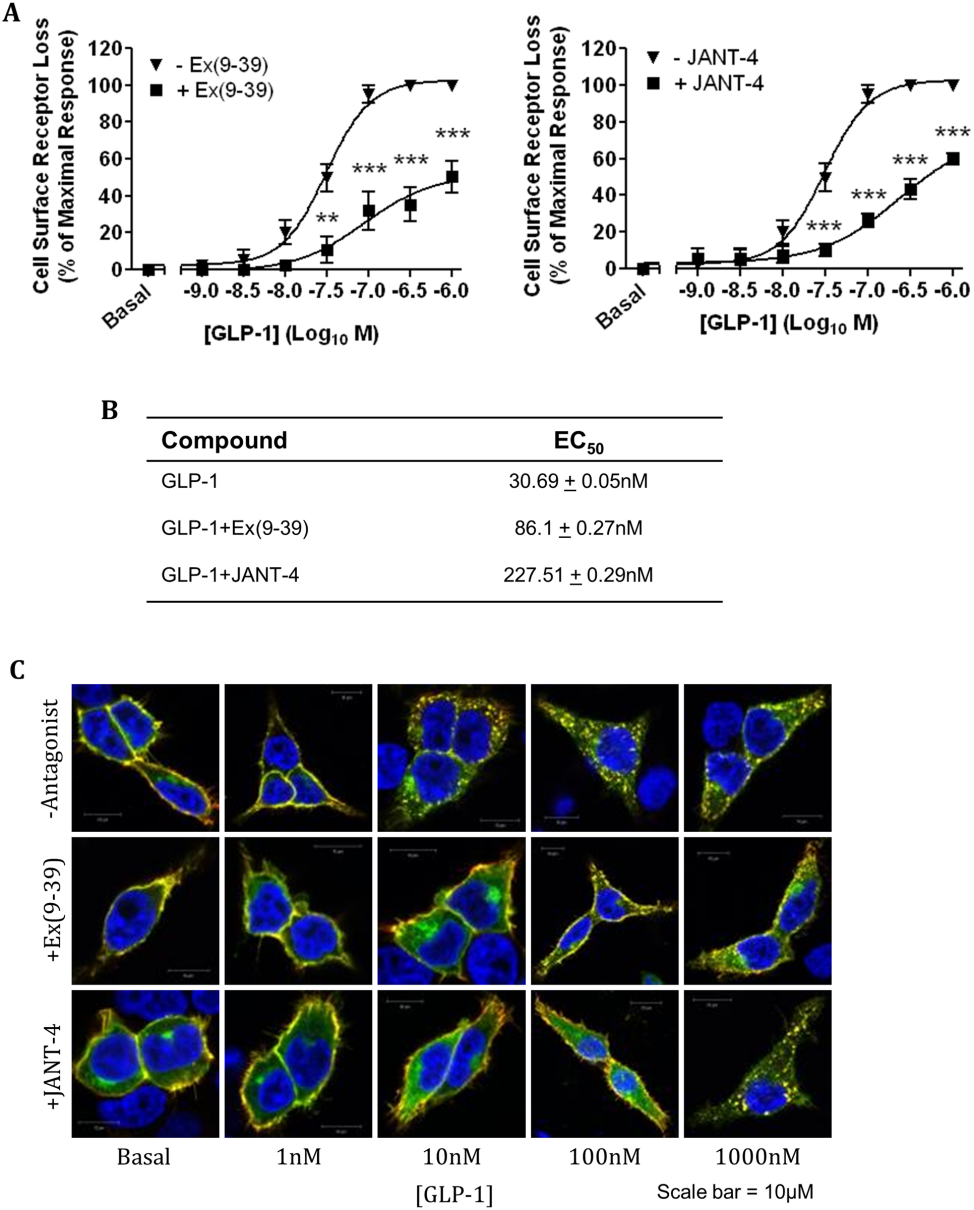
Concentration dependent stimulation of hGLP-1R internalisation by GLP-1 in the presence of antagonists Ex(9–39) and JANT-4. HEK293 cells expressing the hGLP-1R were stimulated with GLP-1 at the indicated concentration for 60min in the presence of 100nM Ex(9–39) (left panel) or JANT-4 (right panel) and the internalisation of hGLP-1R was assessed by ELISA (A) and immunofluorescence (C) using the anti-GLP-1R antibody. (B) The EC_50_ of GLP-1 in the presence or absence of Ex(9–39) or JANT-4 for hGLP-1R internalisation. In immunofluorescence, GFP-tag of hGLP-1R (green) and the anti-GLP-1R antibody staining (red) overlay is shown in yellow and nuclear staining with DAPI in blue. Data are mean + SEM, n = 3, ** p<0.01; *** p <0.001.

Additionally, the antagonists, Ex(9–39) and JANT-4, effect on GLP-1 induced hGLP-1R internalisation was assessed by ELISA and immunofluorescence. In ELISA, the addition of either Ex(9–39) or JANT-4 significantly reduced GLP-1 induced receptor internalisation (Fig 5A) and thereby increased the EC_50_ value of internalisation (Fig 5B). Immunofluorescence analysis supported these observations by demonstrating the inhibition of GLP-1 induced hGLP-1R internalisation by Ex(9–39) and JANT-4 antagonists (Fig 5C). Taken together, these results demonstrate antagonists Ex(9–39) and JANT-4 competitively inhibit hGLP-1R activation by GLP-1 but not by compounds 2 or B, confirming they act through a binding site or binding sites distinct to the orthosteric binding site on the GLP-1R [34].

**Fig 6:**
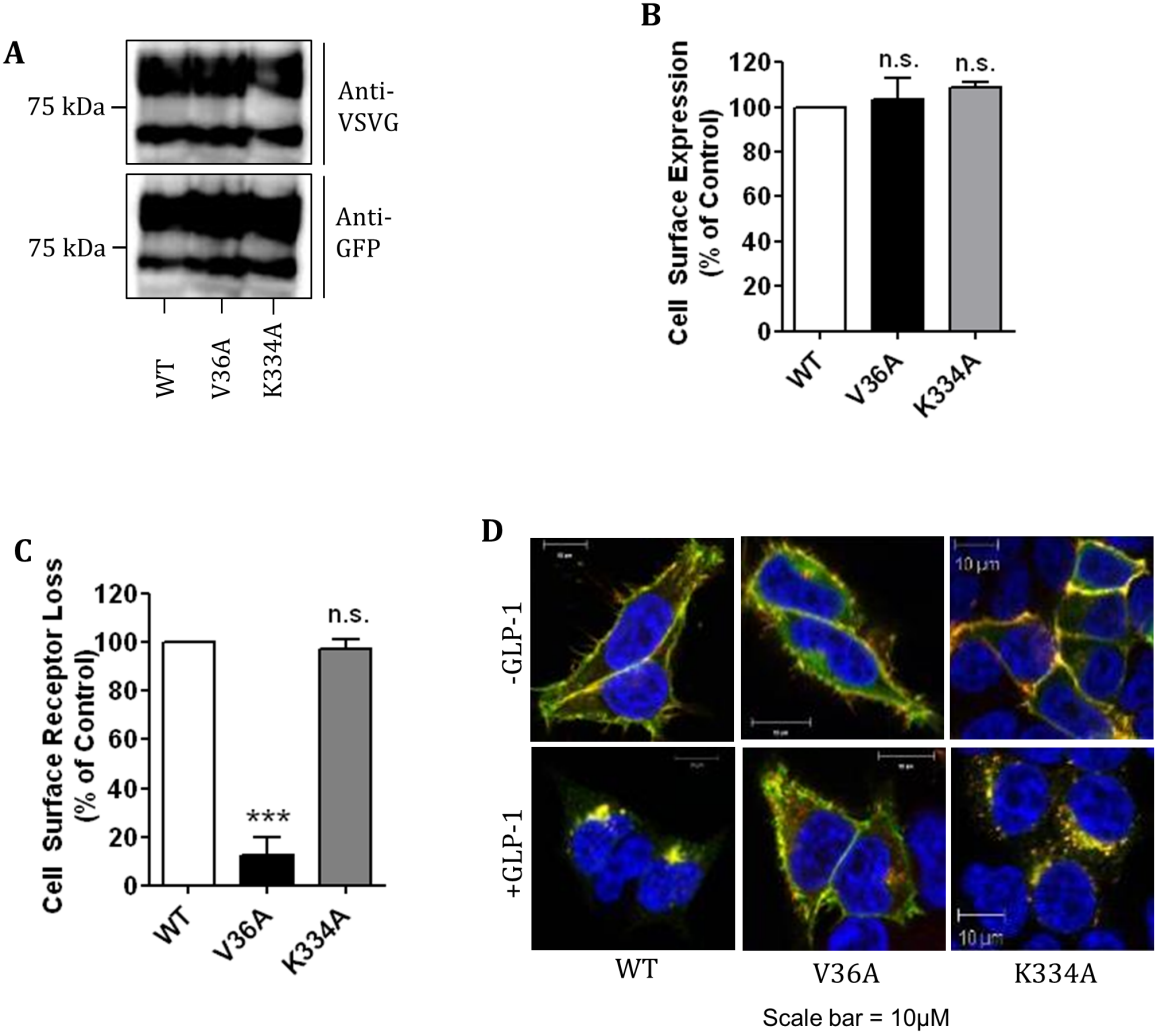
Effect of V36A and K334A mutations of the hGLP1R on the receptor cell surface expression and activity. HEK293 cells were transfected with the hGLP-1R WT (wild-type) or the V36A or K334A mutants for 48h. (A) Total protein expression was assessed by immunoblotting using the anti-GFP and anti-VSVG antibodies. Cell surface expression (B) and agonist induced internalisation (C) were assessed by ELISA using the anti-GLP-1R antibody. (D) Immunofluorescence showing agonist induced internalisation of the WT or mutant hGLP-1R, GFP-tag of the constructs (green) and the anti-GLP-1R antibody staining (red) overlay is shown in yellow and nuclear staining with DAPI in blue. Data are mean + SEM, n = 3, n.s. p<0.05; *** p <0.001.

**Fig 7:**
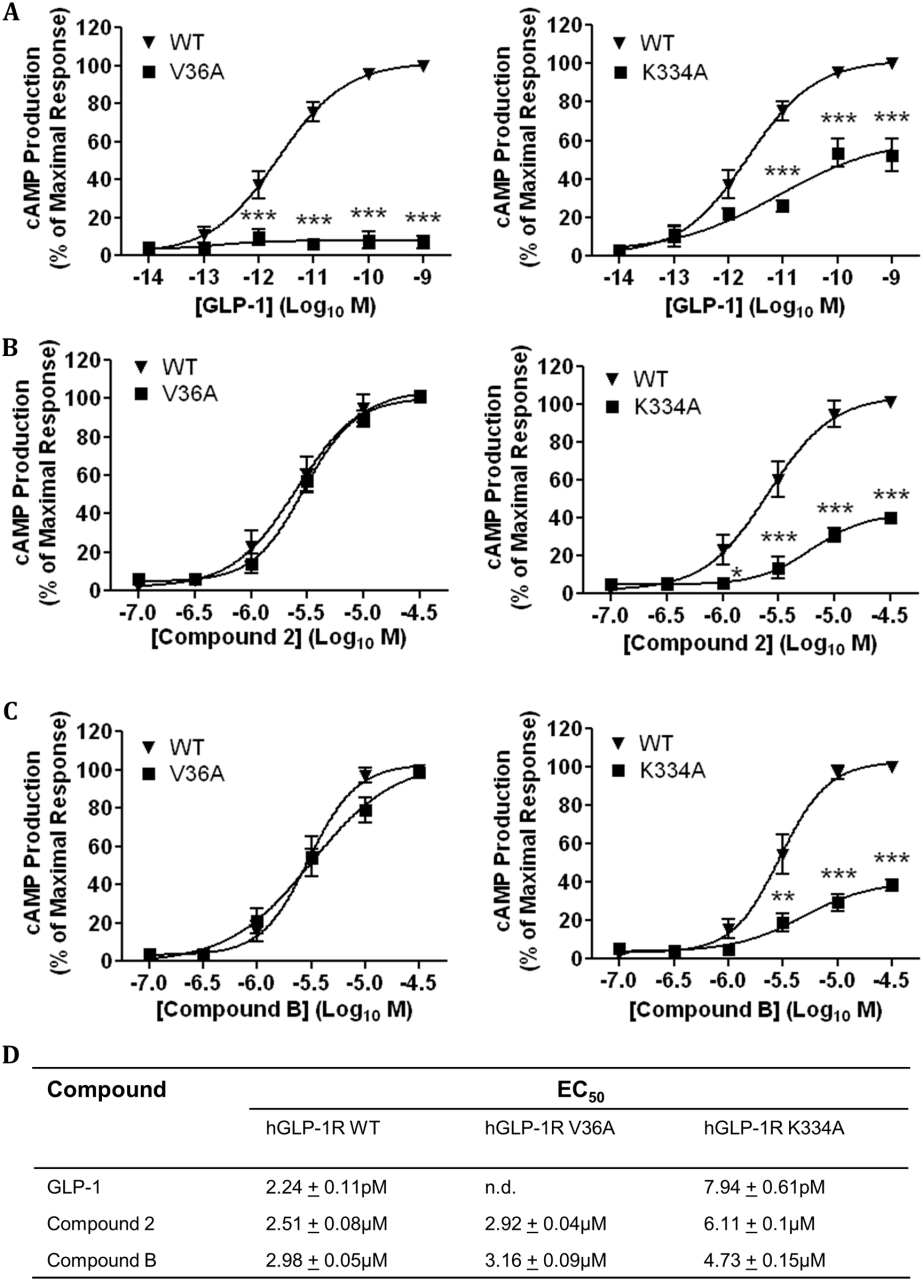
Effect of V36A and K334A mutations of the hGLP-1R on the receptor induced cAMP production. HEK293 cells cotransfected with the V36A (left panel) or K334A (right panel) hGLP-1R plasmid and the luciferase reporter plasmid for cAMP (pGL4.29-Luc-CRE) were stimulated with GLP-1 (A), compound 2 (B) or compound B (C) as indicated to assess cAMP production. (D) The EC_50_ of GLP-1, compounds 2 and B for cAMP production in cells expressing hGLP-1R WT or V36A or K334A (n.d., not determined). Data are mean ± SEM, n = 3, * p<0.05; ** p<0.01; *** p <0.001.

The idea that compound 2 and compound B act through a binding site distinct from the orthosteric binding site was further assessed by using two hGLP-1R mutants (V36A and K334A). The V36A mutant of hGLP-1R prevents agonists binding to the orthosteric binding site [35] whereas the K334A mutant reduces cAMP production by inhibiting the receptor coupling to Gα_s_ subunit [36, 37]. The V36A and K334A mutants were assessed for their expression at protein level (determined by immunoblotting (Fig 6A)), cell surface expression and agonist induced internalisation (determined by ELISA (Fig 6B and 6C) and immunofluorescence (Fig 6D)). The V36A and K344A total protein expression and cell surface expression was similar to that of the wild type (WT) hGLP-1R (103.2 ± 9.55% and 108.9 ± 2.17%, p>0.05, respectively). As expected, agonist induced hGLP-1R internalisation was almost abolished by the V36A mutation (12.4 ± 7.27%, p<0.001) but unaffected by the K334A mutation (97.54 ± 3.67%, p>0.05) [35–37]. HEK293 cells expressing either the hGLP-1R WT, V36A mutant or K334A mutant were treated with increasing concentrations of GLP-1 (Fig 7A), compound 2 (Fig 7B) and compound B (Fig 7C), assessed for cAMP production and calculated EC_50_ values (Fig 7D). GLP-1 increased cAMP production in a concentration dependent manner in the WT expressing cells but not in the V36A mutant (p<0.001) expressing cells. Compound 2 stimulated cAMP production in a concentration dependent manner in both the WT and V36A mutant expressing cells. Compound B also stimulated similar cAMP production in the WT and V36A mutant expressing cells. These results confirmed that the V36A mutation affects the orthosteric binding site of the hGLP-1R. Stimulation of cAMP production in the K334A mutant expressing cells was significantly reduced with GLP-1, compound 2 or compound B treatment. These results suggest that, although the small molecule agonists bind at a different site on the hGLP-1R, GLP-1, compound 2 and compound B may alter conformation of the receptor in a similar way so that the receptor couples to Gα_s_ and induces cAMP production.

### Effect of compounds 2 and B on GLP-1 induced GLP-1R activation and internalisation

**Fig 8:**
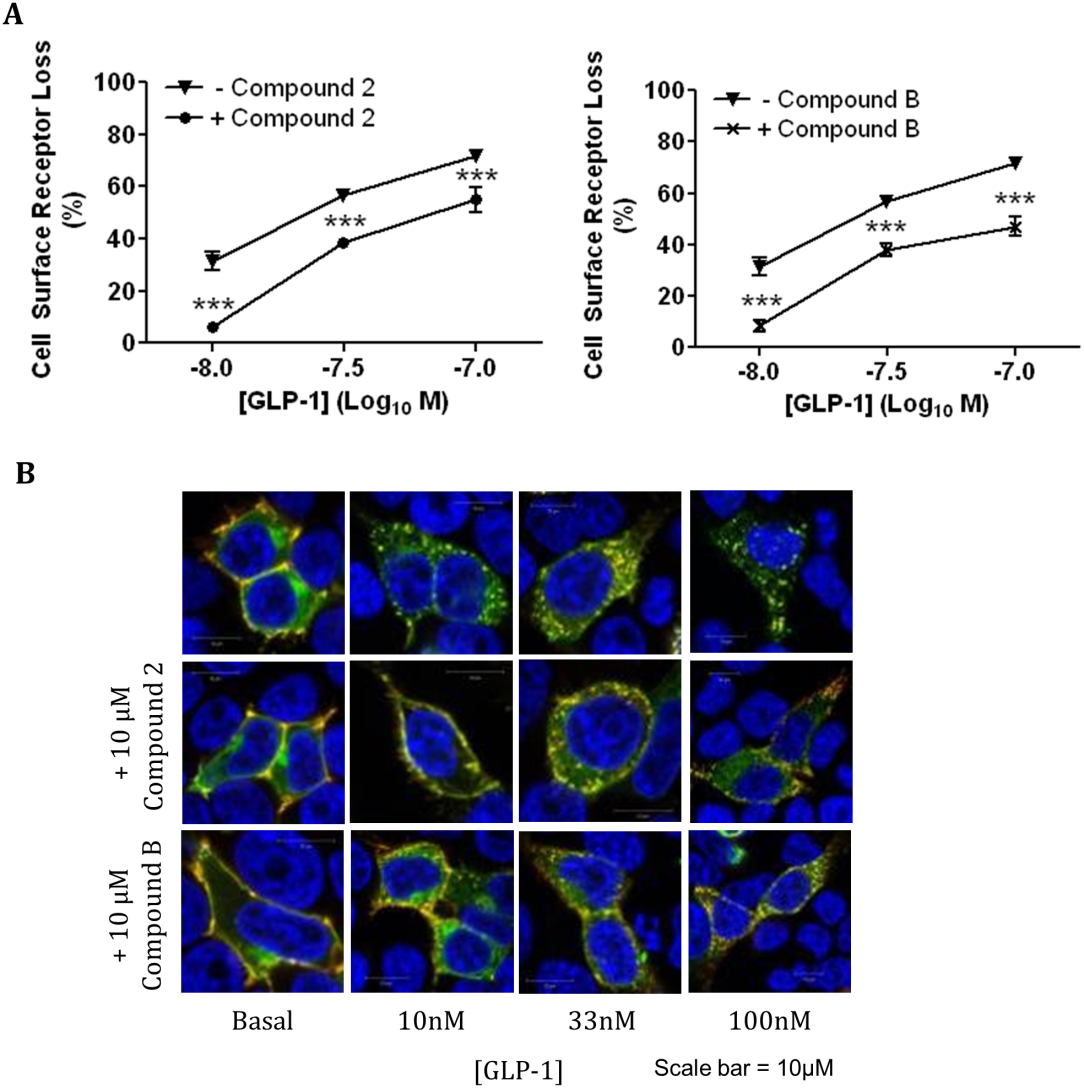
Compounds 2 and B reduce the hGLP-1R internalisation by GLP-1. HEK293 cells expressing the hGLP-1R were pre-incubated with either 10μM compound 2 or B for 60min and then stimulated with GLP-1 at the indicated concentration for a further 60min in the presence of 10μM compound 2 (left panel) or compound B (right panel) and hGLP-1R internalisation was assessed by ELISA (A) and immunofluorescence (B) using the anti-GLP-1R antibody. In immunofluorescence, GFP-tag of hGLP-1R (green) and the anti-GLP-1R antibody staining (red) overlay is shown in yellow and nuclear staining with DAPI in blue. Data are mean ± SEM, n = 3, *** p <0.001.

HEK293 cells expressing the hGLP-1R were pre-incubated with 10μM compound 2 or compound B and then stimulated with increasing concentrations of GLP-1 and internalisation of the receptor was investigated by ELISA (Fig 8A) and immunofluorescence (Fig 8B). Interestingly, compounds 2 and B reduced hGLP-1R internalisation induced with 10nM GLP-1 from 31.34 ± 3.38% to just 6.32 ± 1.25% (p<0.001) and 8.42 ± 2.23% (p<0.001), respectively. The pre-incubation with these compounds also resulted in significant inhibition of internalisation of the receptor induced with 33nM GLP-1 (56.87 ± 1.45% to 38.3 ± 1.83% in the presence of compound 2 and 37.93 ± 2.38% in the presence of compound B, p<0001). Even with the addition of 100nM GLP-1, a significant decrease in hGLP-1R internalisation was observed with compound 2 and compound B pre-incubation (71.78 ± 2.42% with GLP-1 to 55.01 ± 4.81% and 47.02 ± 3.75%, p<0001, respectively). This was further confirmed by immunofluorescence (Fig 8B).

**Fig 9:**
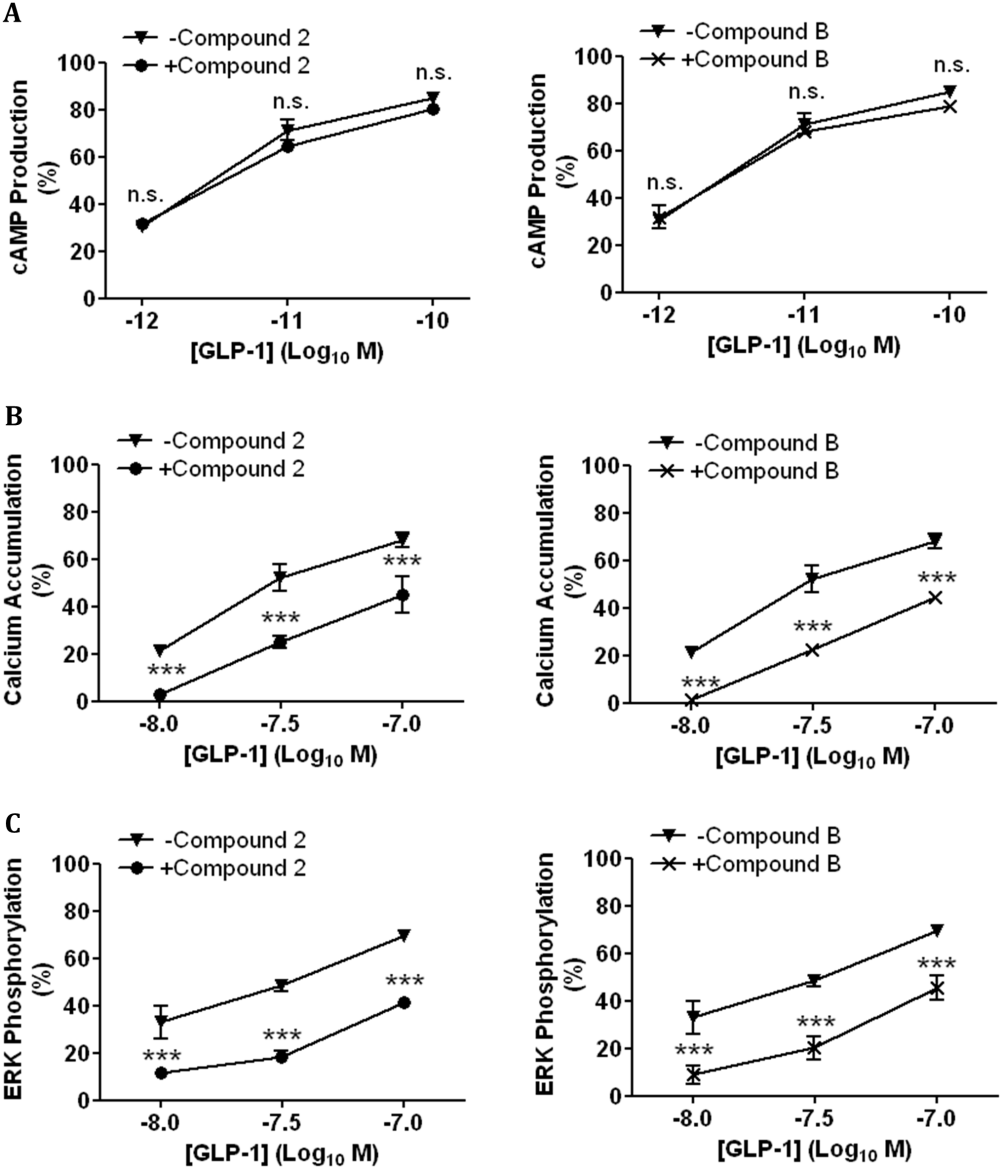
Compounds 2 and B reduce GLP-1 stimulated intracellular Ca^2+^ accumulation and ERK phosphorylation in hGLP-1R expressing cells. HEK293 cells cotransfected with the hGLP-1R plasmid and the luciferase reporter plasmid for cAMP (pGL4.29-Luc-CRE), intracellular Ca^2+^ (pGL4.30-Luc-NFAT) or ERK phosphorylation (pGL4.33-Luc-SRE) were pre-incubated with either 10μM compound 2 or B for 60min. Cells were then stimulated with GLP-1 at the indicated concentrations in the presence of 10μM compound 2 (left panel) or compound B (right panel) to assess cAMP production (A), intracellular Ca^2+^ accumulation (B) and ERK phosphorylation (C). Data normalised to percentage stimulation of GLP-1 and are shown as mean ± SEM, n = 3 (n.s. p>0.05; *** p <0.001).

As pre-incubation with compounds 2 and B reduced GLP-1 induced hGLP-1R internalisation, the effect of compound 2 or B pre-incubation on GLP-1 induced cAMP production (Fig 9A), intracellular Ca^2+^ accumulation (Fig 9B) and ERK phosphorylation (Fig 9C) was assessed. Both small molecule agonists showed no significant effect on GLP-1 induced cAMP production. Interestingly, compound 2 and compound B significantly reduced intracellular Ca^2+^ accumulation with 10nM GLP-1 from 21.42 ± 1.92% to 3.03 ± 1.75% (p<0.001) and 1.13 ± 1.13% (p<0.001), respectively. The addition of 33nM GLP-1 to cells preincubated with 10μM compound 2 or compound B also resulted in significant inhibition of intracellular Ca^2+^ accumulation (52.33 ± 5.6% to 25.17 ± 2.57% and 22.45 ± 0.37%, p<0001, respectively). Even with the addition of 100nM GLP-1, a significant decrease in intracellular Ca^2+^ accumulation was observed with compound 2 and compound B pre-incubation (68.32 ± 2.98% to 45.12 ± 7.92% and 44.75 ± 2.19%, p<0001, respectively).

The pre-incubation of compounds 2 and B also significantly reduced GLP-1 induced ERK phosphorylation. Addition of 10nM GLP-1 to cells induced 33.08 ± 6.99% of maximal ERK phosphorylation, which reduced by pre-incubation with either compound 2 (11.77 ± 1.59%) or compound B (8.99 ± 3.91% [p<0001]). The addition of 33nM GLP-1 to cells pre-incubated with compounds 2 and B reduced ERK phosphorylation from 48.59 ± 2.5% to 18.33 ± 2.51% and 20.2 ± 4.95% (p<0001) respectively. ERK phosphorylation induced by 100nM GLP-1 was also significantly reduced by pre-incubation with compounds 2 or B (70.01 ± 0.46% to 41.59 ± 2.14% and 45.62 ± 5.05%, p<0001, respectively). These results suggest compounds 2 and B either bind the hGLP-1R and cause a conformational change that either reduces GLP-1 access to the orthosteric binding site in a non-competitive manner or prevents GLP-1 occupied GLP-1R coupling to the Gα_q_ pathway and thereby inhibit intracellular Ca^2+^ accumulation or ERK phosphorylation required for hGLP-1R internalisation.

## Discussion

Allosteric small molecule agonists have the potential benefit of binding to a site on the receptor that is distinct from the site used by the orthosteric agonist. Therefore, allosteric agonists can act upon the receptor at the same time as the endogenous orthosteric agonist and alter affinity and/or efficiency of the orthosteric agonist, potentially providing more ‘physiological’ regulations [38]. Recently, two small molecule agonists, compound 2 and compound B, have been described, which act not only as allosteric modulators but also as agonists for the GLP-1R [17, 18]. However, compounds 2 and B shown to have no effect on GLP-1R internalisation, which is mediated by the Gα_q_ pathway [20]. In this study, we pharmacologically assessed their effect on GLP-1R activity and internalisation.

In agreement with our previous study [20], both the small molecule agonists of the hGLP-1R induced cAMP production but not intracellular Ca^2+^ accumulation or ERK phosphorylation and as a result they did not induce hGLP-1R internalisation. Compounds B and 2 have previously been reported to induce a small or no increase in intracellular Ca^2+^ accumulation [4, 25, 39], which effect, however, on GLP-1R internalisation is unknown. Studying the internalisation of GLP-1R induced by compounds 2 and B is useful in assessing the effectivity of these compounds with longer half-life. This is because internalisation of the receptor can lead to dampening of its biological response [32]. The μ-opioid receptor agonist, herkinorin, induces ERK1/2 phosphorylation but not internalisation of the receptor [40]. Additionally, the allosteric agonist AC-42 binds to the M_1_ muscarinic acetylcholine receptor, which results in ERK phosphorylation and intracellular Ca^2+^ mobilisation but not internalisation of the receptor [41, 42]. This suggests orthosteric and allosteric agonists can cause subtle differences in the conformation of the receptors, activating separate signalling pathways. Additionally, this further supports the idea that the GLP-1R does not require cAMP for internalisation of the receptor, but instead intracellular Ca^2+^ accumulation and ERK phosphorylation are essential [20].

In this study, antagonists Ex(9–39) [9, 10] and JANT-4 [26] inhibited GLP-1 induced GLP-1R internalisation and signalling but not compound 2 or compound B induced signalling, suggesting a second agonist binding site on the hGLP-1R that is distinct from the orthosteric binding site. These findings are consistent with the results obtained in previous studies for compound 2 [17] and compound B [17, 18], which showed antagonist Ex(9–39) had no effect on cAMP signalling. This is further confirmed by using two mutants of the hGLP-1R (V36A and K334A). The V36A mutation in the GLP-1R has previously been shown to affect GLP-1 binding to the orthosteric binding site [35]. In this study, HEK293 cells expressing the V36A mutant did not show GLP-1 stimulated cAMP. In contrast, the V36A mutant expressing cells did show compounds 2 and B stimulated cAMP production to the same levels produced in the hGLP-1R expressing cells upon exposure to these compounds. These results demonstrated that the V36A mutation in the hGLP-1R only affects the orthosteric binding site and, compounds 2 and B interact with the hGLP-1R at a site different to the orthosteric binding site. Additionally, the K334A mutation in the GLP-1R, which has previously been shown to prevent coupling of the receptor to the Gα_s_ subunit [36, 37], reduced cAMP production stimulated by GLP-1, compound 2 and compound B. This demonstrates that these small molecule agonists and GLP-1 induce similar conformational changes in the hGLP-1R, which are required for the Gα_s_ coupling, although they bind at different sites on the hGLP-1R. While this manuscript was being prepared, a recent study showed that compounds 2 and B covalently modifies Cys347 in intracellular loop 3 of GLP-1R [34].

In this study, compounds 2 and B have been shown to reduce GLP-1 induced hGLP-1R internalisation, intracellular Ca^2+^ accumulation and ERK phosphorylation. Reducing hGLP-1R internalisation prevents dampening of the receptor activity [43]. Therefore, these small molecule agonists may strengthen GLP-1 potency by allowing the orthosteric agonist to act on the receptor for a prolonged period before it is desensitised. Furthermore, they have also been shown to allosterically modulate the function of GLP-1 [9–36]-NH_2_ (GLP-1 metabolite) by sensitising the GLP-1R for activation by this metabolite [18, 34, 44, 45]. Based on this ability, compounds 2 and B may provide insight into the mechanisms of agonist directed GLP-1R regulation and may represent a step further in the development of effective insulinotropic agents with limited adverse effects. This result is similar to the effect of allosteric agonists of the cannabinoid CB_1_ receptor, because their binding to the receptor results in a conformation change that increases the affinity of the orthosteric agonist to the receptor [46]. In contrast to compounds 2 and B, allosteric agonist alcuronium inhibits the actions of orthosteric agonist pilocarpine on the M_2_ muscarinic acetylcholine receptor [47]. It has also been shown that compounds 2 and B binding cause a conformational change in GLP-1R that increases GLP-1 access to the orthosteric binding site [17, 18]. Therefore, it is possible that they prevent GLP-1 induced GLP-1R internalisation by inhibiting GLP-1 bound hGLP-1R coupling to the Gα_q_ pathway required for intracellular Ca^2+^ accumulation and thereby ERK phosphorylation [20]. It has been shown that the intracellular C-terminal domain of GLP-1R is important for agonist-induced internalisation of the receptor [23]. Furthermore, the agonist induced GLP-1R internalisation has recently been shown to be important for insulin secretion [48]. However, compounds 2 and B not only induce insulin secretion but also inhibit GLP-1R internalisation [17, 18, 20], indicating that GLP-1R may use multiple and compensatory pathways to induce insulin secretion in pancreatic islets.

**Fig 10:**
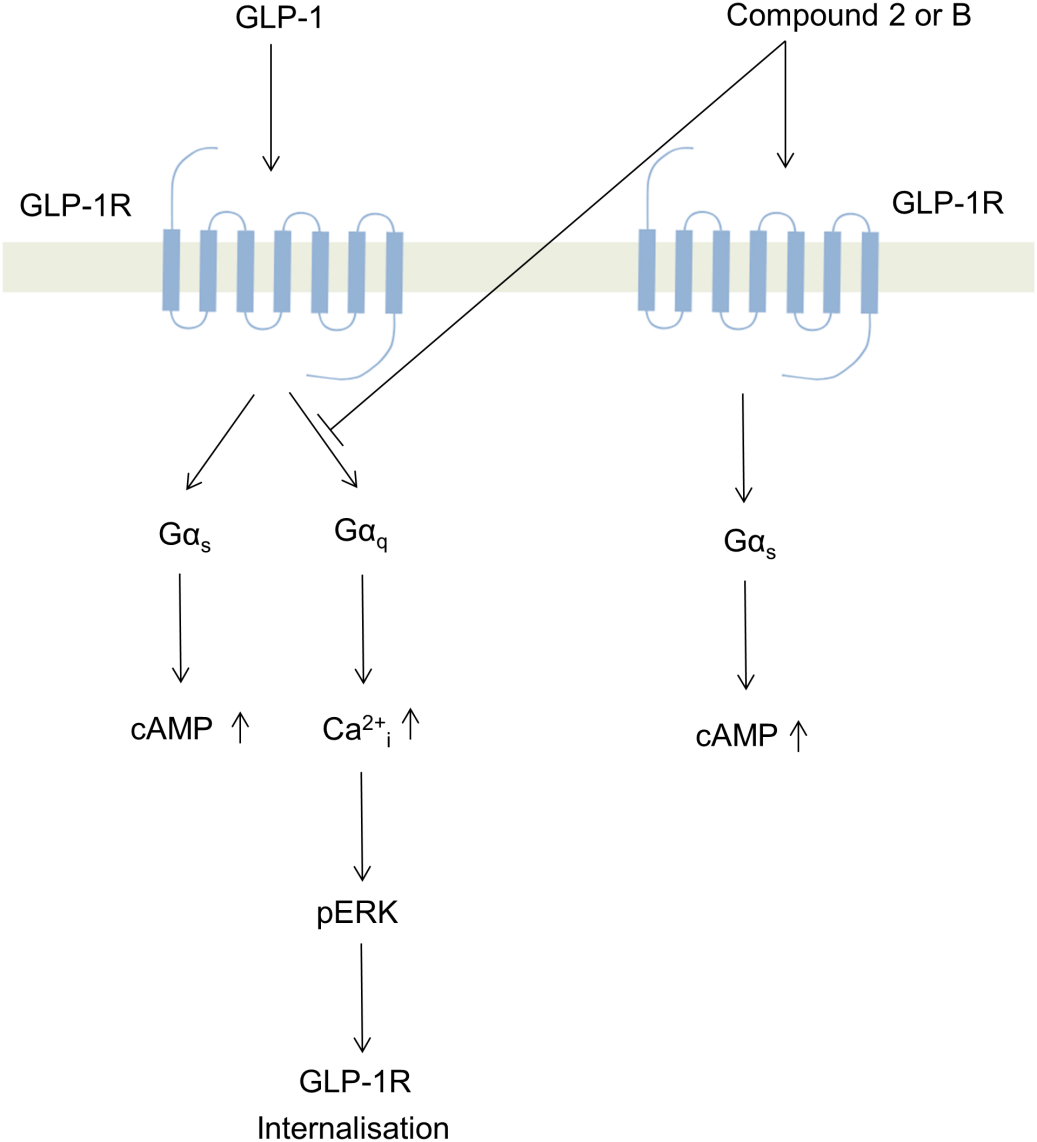
Schematic representation of the downstream signalling pathways activated by GLP-1 and small molecule agonist compounds 2 and B deduced from this study (Ca^2+^_i_, intracellular Ca^2+^ accumulation).

In summary, small molecule agonists, compound 2 and compound B, were pharmacologically analysed in this study for their effect on hGLP-1R internalisation, cAMP production, intracellular Ca^2+^ accumulation and ERK phosphorylation. Although the small molecule agonists induced cAMP production with similar E_max_ to GLP-1, they did not induce intracellular Ca^2+^ accumulation and ERK phosphorylation (mediated by the Gα_q_ pathway) and as a result these agonists did not induce hGLP-1R internalisation (Fig 10). We have recently shown that agonist induced internalisation of the GLP-1R is mediated by the Gα_q_ pathway [20]. With the use of antagonists and the V36A mutant of the hGLP-1R, this study demonstrated that compounds 2 and B act on a region of the hGLP-1R independent to the orthosteric agonist site. However, the use of the K334A mutant of the hGLP-1R demonstrated that compounds 2 and B induce a conformational change in the GLP-1R, which is required for the Gα_s_ coupling, similar to that induced by the orthosteric agonist binding to the receptor. Additionally, compounds 2 and B reduced, in a non-competitive manner, GLP-1 induced GLP-1R coupling to Gα_q_ and internalisation (Fig 10). Therefore, this study suggests a potential advantage in the selective activation of specific signalling pathways (biased agonism) by ago-allosteric agonists compounds, which may cause changes in GLP-1R conformation that are less favourable to the internalisation of the receptor.
